# Desiccation limits recruitment in the pleometrotic desert seed‐harvester ant *Veromessor pergandei*

**DOI:** 10.1002/ece3.7039

**Published:** 2020-11-22

**Authors:** Robert A. Johnson

**Affiliations:** ^1^ School of Life Sciences Arizona State University Tempe AZ USA

**Keywords:** cooperation/grouping, environmental harshness, foundress ants, moisture, seed‐harvester ants, Sonoran Desert

## Abstract

The desert harvester ant *Veromessor pergandei* displays geographic variation in colony founding with queens initiating nests singly (haplometrosis) or in groups (pleometrosis). The transition from haplo‐ to pleometrotic founding is associated with lower rainfall. Numerous hypotheses have been proposed to explain the evolution of cooperative founding in this species, but the ultimate explanation remains unanswered. In laboratory experiments, water level was positively associated with survival, condition, and brood production by single queens. Queen survival also was positively influenced by water level and queen number in a two‐factor experiment. Water level also was a significant effect for three measures of queen condition, but queen number was not significant for any measure. Foundress queens excavated after two weeks of desiccating conditions were dehydrated compared to alate queens captured from their natal colony, indicating that desiccation can be a source of queen mortality. Long‐term monitoring in central Arizona, USA, documented that recruitment only occurred in four of 20 years. A discriminant analysis using rainfall as a predictor of recruitment correctly predicted recruitment in 17 of 20 years for total rainfall from January to June (the period for mating flights and establishment) and in 19 of 20 years for early plus late rainfall (January–March and April–June, respectively), often with a posterior probability > 0.90. Moreover, recruitment occurred only in years in which both early and late rainfall exceeded the long‐term mean. This result also was supported by the discriminant analysis predicting no recruitment when long‐term mean early and late rainfall were included as ungrouped periods. These data suggest that pleometrosis in *V. pergandei* evolved to enhance colony survival in areas with harsh abiotic (desiccating) conditions, facilitating colonization of habitats in which solitary queens could not establish even in wet years. This favorable‐year hypothesis supports enhanced worker production as the primary advantage of pleometrosis.

## Graphical abstract

This manuscript addresses abiotic physiological tolerance of ant queens during colony founding, suggesting that grouping facilitates survival during favorable years when single queens could not survive. Moreover, grouping by ant queens may facilitate colonizing harsh environments in a manner similar to that suggested for cooperative breeding birds.

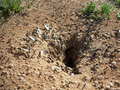

## 1 INTRODUCTION

Water is the primary factor controlling ecological processes in deserts, including demographic patterns, distribution patterns, physiological processes, response thresholds, and life history attributes of a species (Ehleringer & Sandquist, 2018; Johnson, 2006; Jordan & Nobel, 1979; Nobel, 1988; Noy‐Meir, 1973; Reynolds et al., 2004; Riddell et al., 2019). These processes are affected by amount of rainfall as well as by high interannual variability and unpredictability in rainfall (MacMahon & Wagner, 1985) as evidenced by changes in occurrence and microdistribution of species following decadal changes in rainfall (Ehleringer & Sandquist, 2018; Iknayan & Beissinger, 2018). Recruitment of new individuals in desert habitats also is intimately linked to rainfall for many species (Gibson & Nobel, 1986; Nobel, 1988).

Moisture is one of the most limiting abiotic factors for invertebrates because their small size and high surface‐to‐volume ratios make them prone to desiccation (Hadley, 1994; Harrison et al., 2012). Evidence of such limitation most frequently involves studies that correlate physiological tolerances of organisms with their microdistribution pattern (Bujan et al., 2016; Chown & Klok, 2003; Hood & Tschinkel, 1990; Talbot, 1934). Water loss rate, dehydration tolerance, and body size combine to determine physiological tolerance of organisms to desiccation. In arid habitats, increased body size appears to be one of the more important characters related to increasing desiccation resistance (Chown & Klok, 2003; Le Lagadec et al., 1998; Scholtz & Caveney, 1988; Tejeda et al., 2016). Insects and other arthropods can also use behaviors such as grouping to increase their effective body size, thus resulting in physiological benefits that include decreased water loss and increased longevity (Bong et al., 2018; Broly et al., 2014; Klok & Chown, 1999). As a result, grouping has potential to allow organisms to inhabit more physiologically harsh areas than possible by solitary individuals (Avilés, 1999; Liu et al., 2020).

Colony founding is the most critical and vulnerable stage in the life of ant colonies, and this is also the stage at which desiccation would have its strongest effect on limiting recruitment of new colonies (Hölldobler & Wilson, 1990; Johnson, 1998). Studies that directly assess the effect of moisture on founding queens demonstrate a positive correlation between moisture and queen survival, queen condition, and number of first brood workers (Cahan, 1999; Johnson, 1998). Body size also affects desiccation tolerance and microhabitat occurrence for two other desert ant species in the genus *Pogonomyrmex*; the larger queens of *P. rugosus* are more desiccation tolerant than those of *P. barbatus*, which correlates with *P. rugosus* colonies occurring in more xeric microhabitats than those inhabited by *P. barbatus* (Johnson, 2000a). In some ant species, multiple unrelated queens group during colony founding (pleometrosis) (Bernasconi & Strassman, 1999), and this behavior has potential to enhance queen survival and the establishment of new colonies, that is, recruitment, because of physiological benefits similar to those observed for other insects (see above), but data are lacking (but see Cahan, 1999).

The proximate benefits of pleometrosis include at least one of the following: (a) earlier emergence of first workers, (b) increased number of workers, (c) decreased mass loss by queens, (d) increased defense against workers from neighboring mature colonies, and (e) disease control (Bartz & Hölldobler, 1982; Jerome et al., 1998; Pull & Cremer, 2017; Rissing & Pollock, 1987; Sommer & Hölldobler, 1995; Tschinkel & Howard, 1983). Pleometrosis can also enhance the survival rate of queens, though the mechanism causing this increase is unclear (Johnson, 2004; Waloff, 1957). At the ultimate level, for pleometrosis to evolve and persist requires that survival probability for a queen in an association of *n* queens is more than *n* times greater than that of a solitary queen (Adams & Tschinkel, 1995). Overall, pleometrosis is viewed as advantageous when intercolony competition is intense, both by adult and incipient colonies (for a review see Bernasconi & Strassman, 1999), or under harsh abiotic conditions (Cahan, 2001b; Pfennig, 1995). Harsh abiotic conditions have been suggested to favor pleometrosis because of its potential to confer greater desiccation resistance for groups compared to single queens (Cahan, 2001b). Notably, these two conditions (intense intercolony competition and harsh abiotic environment) correspond to different levels of survival; high levels of founding queen survival support the intercolony competition hypothesis, while low queen survival supports the hypothesis that harsh abiotic conditions limit colony recruitment.

Several studies document that recruitment of new colonies is rare in the desert seed‐harvester ant *Veromessor pergandei* (Mayr) (formerly *Messor pergandei*) (Pfennig, 1995; Raczkowski, 2003; Ryti & Case , 1988). The several mostly proximate hypotheses presented to explain the evolution of pleometrosis in this species include: (a) intraspecific competition in the form of higher success at brood raiding (Rissing & Pollock, 1987), (b) inter‐colony interference competition (Pfennig, 1995), (c) the need to quickly find a nest to avoid desiccation and predators (Pfennig, 1995), (d) enhanced foraging success leading to increased colony survival and growth (Brown, 2000), and (e) enhanced water balance of grouped compared to solitary queens, especially under xeric conditions (Cahan, 1999; Raczkowski, 2003).

Two field studies have examined pleometrosis in *V. pergandei* with contradictory results. Pfennig (1995) found no difference in survival or longevity for one versus two queen colonies, leading him to suggest that interference competition among incipient colonies does not select for pleometrosis. Alternatively, at the same site Raczkowski (2003) found that both survival and longevity were higher for three queen compared to one queen colonies, and that distance to a mature colony did not affect longevity.

This study tests hypotheses related to founding queen survival and recruitment of new colonies in a pleometrotic population of *V. pergandei*. I used laboratory experiments, field data, and long‐term census data to test the hypothesis that insufficient moisture limits survival of founding queens and recruitment of new colonies. I also tested the desiccation resistance hypothesis using a two‐factor (queen number × moisture level) laboratory experiment to determine the relative importance of these two effects on queen survival and condition. This experiment also tested two predictions of the desiccation resistance hypothesis: (a) grouping enhances queen survival to a higher degree under desiccating compared to mesic conditions, and (b) a more positive water balance results for grouped than for solitary queens under desiccating conditions.

## 2 METHODS

### 2.1 Study species

*Veromessor* *pergandei* (subfamily myrmicinae) is a common soil nesting, seed‐harvester ant that inhabits the Sonoran, Colorado, and Mojave Deserts, typically in sandy soils at elevations < 900 m—areas that collectively encompass the hottest, most arid portions of North America (Creighton, 1950, 1953; Johnson, 1992, 2000c; Tevis, 1958). Mature colonies contain > 35,000 foragers that form conspicuous foraging trails that extend > 35 m from the nest (Plowes et al., 2012).

The colony cycle of *V. pergandei* begins with mating flights, which usually occur from mid‐February to late March (Cahan, 2001b; Ode & Rissing, 2002; Pollock & Rissing, 1985). A moderate number of sexuals fly from nests on each flight day, with sexuals flying 30–50 m above ground to mate in the air (i.e., non‐lek mating); pairs sometimes fall to the ground *in copulo*. Founding queens initiate nests throughout a site, though they are most common in open areas that lack vegetation (Rissing & Pollock, 1989).

Queen founding behavior varies across the geographic range: single queens (haplometrosis) initiate nests in western portions of the range, that is, southwestern Arizona and southern California, whereas multiple, unrelated queens (pleometrosis) initiate nests in eastern and northwestern portions of the range, that is, central and western Arizona, southeastern California, and southern Nevada (Hagen et al., 1988; Helms Cahan & Helms, 2012; Johnson, 2000b; Pollock & Rissing, 1985; Rissing et al., 2000; Ryti, 1988). In areas of pleometrosis, queens accumulate in founding nests during the mating season, but additional queens are never accepted in the colony after the first workers emerge. Pleometrotic queens cooperate to produce the first brood of workers, with worker production being a linear function of queen number (Rissing & Pollock, 1986, 1991). Additional variation occurs in pleometrotic parts of the range because colonies reduce to one queen (monogyny) after worker emergence in eastern areas, whereas multiple queens persist in mature colonies (primary polygyny) in northwestern portions of the range (Helms & Helms Cahan, 2012; Rissing & Pollock, 1986, 1987). In southeastern California, the shift from haplometrosis to pleometrosis occurs across a narrow transition zone with the shift to pleometrosis correlating with reduced precipitation, decreased vegetative biomass, and lower colony density (Cahan, 2001a, 2001b; Cahan et al., 1998). Surviving colonies then enter their growth phase and probably start producing reproductive sexuals after 3–4 years; colonies likely live 10–20 + years (Tevis, 1958; Wheeler & Rissing, 1975).

### 2.2 Study site

I studied *V. pergandei* at a site that extended from approximately 2 km south to 3 km southwest of Signal Peak, Pinal County, Arizona (32^o^56′–57′N, 111^o^40′–42′W; elevation 435–450 m). Habitat of the area was typical Sonoran Desert and consisted of a creosote bush (*Larrea tridentata*)—triangle leaf bursage (*Ambrosia deltoidea*) association with scattered ironwood (*Olneya tesota*) and foothill palo verde (*Parkinsonia microphyllum*). Substrate consisted of moderately coarse sand (see Johnson, 1992). Other common ants at the site included *Pogonomyrmex rugosus*, *Pogonomyrmex pima, Pheidole xerophila*, *Dorymyrmex insanus*, and *Solenopsis xyloni*. *Veromessor pergandei* queens initiate nests pleometrotically at this site, but queens reduce to monogyny following emergence of the first workers (Pfennig, 1995; Rissing & Pollock, 1986, 1987).

### 2.3 Laboratory experiments on moisture level and queen number

I conducted two laboratory experiments to examine the effect of moisture on queen survival and brood production. The first experiment examined the effect of moisture level on survival, condition (wet mass, water content, dry mass), and brood production for single queens. The second experiment used a two‐factor design to examine the effects of queen number and moisture level on queen survival and condition.

Both experiments used eight ounce (0.24 L) glass bottles that contained 325 g of soil and 45 mls of water. Soil was collected at the study site in areas occupied by *V. pergandei*, passed through a 2 mm sieve, mixed into a composite sample, weighed, and placed into bottles. The moisture treatments involved adding water to bottles every 10 days starting on day 20; the first experiment used four moisture levels—0, 5, 10, and 15 mls of water; the second experiment used 0, 5, and 10 mls of water. Founding queens were excavated from their incipient nests, placed in closed containers with moist paper towels for several hours, weighed, and then placed in bottles. Bottles in the first experiment contained one queen (*n* = 270; 100 in 0 mls water, 70 in 5 mls water, 50 in 10 mls water, 50 in 15 mls water). Bottles in the second experiment contained one, three, or five queens (*n* = 900 queens in 460 bottles; 120 queens per queen treatment in 0 mls water, 120 queens per queen treatment in 5 mls water, 60 queens per queen treatment in 10 mls water); queens in the second experiment were uniquely marked with Tamiya paint (Aliso Viejo, California) prior to weighing. Both experiments were set up so that initial wet mass of queens was similar across treatments (experiment 1: one‐way ANOVA, *F*_3,266_ = 0.30, *p* > 0.80; experiment 2: *F*_8,891_ = 0.77, *p* > 0.60), thus minimizing effects caused by mass. Bottles were sealed for 1–2 days to allow queens to excavate their nest. I then removed and replaced dead queens that were visible on the soil surface, and lids were replaced with a plastic petri dish that contained several holes. Bottles were maintained in a darkened room at 20–25°C.

In the first experiment, bottles were emptied after 92 days, which was approximately 1–2 weeks after workers began to appear on the soil surface. Bottles were emptied after 66 days in the second experiment; this time interval was chosen so as to end the experiment prior to worker emergence and queen reduction. Queen status (live or dead) was determined in both experiments, and brood (larvae, pupae, workers) were counted in the first experiment. Live queens were weighed, dried for > 72 hr at 50–55°C, and then reweighed.

In both experiments, queen survival was analyzed by a logistic regression that used a binomial distribution and logit link function (PROC GENMOD in SAS 6.12) (SAS Institute, 1997). Queen status (live or dead) was the dependent variable and moisture level was the independent variable in the first experiment; moisture level and queen number were the independent variables in the second experiment. The model uniquely coded each treatment cell in both experiments, which resulted in comparing survival rates across all treatment cells simultaneously. Significance levels were based on the Wald Chi‐square statistic.

In the first experiment, queen wet mass, water mass, dry mass, and number of brood were analyzed across moisture levels using a one‐way ANOVA. In the second experiment, I included data for only bottles in which ≥ 2 queens and ≥ 4 queens survived in the three and five queen bottles, respectively. Wet mass, water mass, and dry mass were each analyzed using a two‐way ANOVA followed by a univariate Duncan's multiple range post hoc test for the two main effects. This was followed by a one‐way ANOVA with Duncan's multiple range test across all cells. Data were transformed, as necessary, to meet the assumptions of ANOVA.

### 2.4 Growth of single queen colonies in the laboratory

I determined number of workers and brood that single queens produced by the end of the founding stage as well as over their first several months given that more workers hasten increasing the size and depth of the nest so as to escape harsh temperature and moisture conditions near the soil surface. I determined number of minim workers produced by the end of the founding stage by placing single queens in glass “ants farms” (16 × 10 × 1.5 cm) filled with soil, which facilitated observing nests and counting minim workers. I placed additional single queens in 12 oz bottles (0.35 L) filled with sieved soil that had been thoroughly moistened; the bottles were then sealed with a screw‐top lid and placed in an incubator at 30 ± 1.0°C. Colonies were given ad libitum Kentucky bluegrass seeds (*Poa pratensis*) after the first workers emerged. Bottles were emptied after 90 days, and workers, pupae, and larvae were counted.

### 2.5 Desiccation of founding queens in the field

I also examined desiccation as a source of mortality for founding queens by comparing water status for alate queens in their natal nests to founding queens excavated from their incipient nests. In 1999, *V. pergandei* had its first mating flight on 19 February, and additional flights were absent to very weak until 1 March, when sexuals exited nests for a flight that was largely aborted. On both dates, I collected 10 unmated alate (winged) queens from outside natal nests at each of three colonies. Founding queens were excavated on 3 March; these queens had likely initiated nests about 2 weeks earlier given the absence of flight activity from 19 February to 3 March. Each alate/founding queen was placed in an Eppendorf tube, placed on ice, and weighed within 15 min of capture on a Mettler AE 163 balance attached to a power inverter. Individuals were then returned to the laboratory, placed in an oven at 50–55°C for > 72 hr, and reweighed.

I assessed water status of field‐weighed alate queens by comparing their water content to that of hydrated alate queens. Determining water content of hydrated queens involved collecting 10 alate queens from each of eight colonies in 1993 and 1994 (four colonies from the study site and four colonies from 5 km east of the White Tank Mountains, Maricopa County, AZ [33^o^36′N, 112^o^28′W; 400 m]). Individuals were placed in an enclosed container with a moistened paper towel and allowed to hydrate for several hours; they were then weighed, dried at 50–55°C for > 72 hr, and reweighed. Wet mass, water mass, and dry mass were compared across the four groups of queens (alates collected on each of two dates, hydrated alates, founding queens) using a one‐way ANOVA followed by Duncan's multiple range test (SPSS, 1990).

I also quantified dehydration tolerance of alate queens so as to determine amount of water loss at which mortality occurs in the field. Dehydration tolerance was determined using hydrated alate queens (3–4 individuals from each of 10 colonies), which were weighed, and individuals were then placed in 20 × 7 mm chambers made of rigid plastic tubes sealed on both ends with push‐fit caps of stainless steel screen (Johnson, 2000d; Johnson et al., 2011). Chambers were connected in tandem with pliable plastic tubing, with up to 10 chambers per column. Individuals were placed at 15–20°C, which mimicked soil temperatures that queens experience during early phases of nest founding. Air desiccated by Drierite was forced through columns at a rate of 100–150 mls/min, as controlled by a needle valve and rotameter. Individuals were examined every few hours; those that could not right themselves were weighed, dried for > 72 hr at 50–55°C, and reweighed. All mass loss over this period was assumed to result from water loss. Dehydration tolerance was calculated using the formula DT = 100 × (*L*/*H*), where DT is the percent body water lost at the time an individual could not right herself, *L* is water mass (mg) lost at the time of losing locomotor ability, and *H* is water mass (mg) in a hydrated state. These data were used to assess hydric condition of founding queens in the field.

### 2.6 Recruitment in the field

Six 1.25 ha (112 × 112 m) plots were established in 1991 about 2.5 km south of Signal Peak (see above). Plots were censused each year between late August and late November, 1991–2001. Censuses were conducted by walking systematic 5 m wide transects throughout each plot, noting colonies of *V. pergandei* that had been initiated earlier that year based on their small nests and absence of workers larger than a minim. These plots were subsequently developed and could no longer be censused. In 2005 and 2012–2019, I surveyed for new colonies of *V. pergandei* by walking through the general area over several days.

Rainfall was examined as a factor limiting recruitment by *V. pergandei* over the 20 surveyed years. The annual rainfall pattern is bimodal in this part of the Sonoran Desert with similar amounts falling in winter and summer (Reynolds et al., 2004). The 1–2 month period prior to mating flights up to the beginning of summer rains, that is, January through June, is viewed as the most critical interval for rainfall to affect survival for founding queens of *V. pergandei*. Moreover, rainfall decreases dramatically near the end of March, and the three spring months—April, May, and June—are the three driest months of the year (MacMahon & Wagner, 1985; Reynolds et al., 2004). Summer rains typically begin in early to mid‐July. This pattern is exemplified by the Casa Grande weather station, which is the station nearest (≈6.5 km SW) to the field site. From January through June, 1981–2009, rainfall at this station averaged 80.77 mm, with 64.01 mm (79.2%) falling from January–March and 16.76 mm (20.8%) from April–June (see http://www.wrcc.dri.edu/cgi-bin/cliMAIN.pl?az1306).

Daily rainfall data were obtained from the National Oceanic and Atmospheric Administration, National Climatic Data Center (https://www.ncdc.noaa.gov/cdo-web/datasets/GHCND/stations/GHCND:USC00021306/detail) for the Casa Grande weather station, which is the station nearest to the study site (see above), or from the Casa Grande National Monument station (≈13 km NE of the site) when data were missing from the Casa Grande station. The Casa Grande weather station ceased operating in 2009. In subsequent years, nearby stations operated for short periods. I used the Casa Grande 4.2 NE station in 2012 (≈6.5 km SW of site), the Casa Grande 1.8 NE station in 2013 (≈7 km SW of site), and the Casa Grande 6.5 NNW station in 2014–2019 (≈10 km NNW of site). For each year, I summed daily rainfall to obtain two measures: (a) total rainfall for January–June, and (b) a measure that separated rainfall into two periods, January–March (early rainfall) and April–June (late rainfall).

I assessed effect of rainfall on recruitment using discriminant analysis (SPSS, 1990). Recruitment was a binomial dependent variable (recruitment or no recruitment) and rainfall was the independent variable. The two rainfall variables, total rainfall and early plus late rainfall, were assessed in separate runs of the discriminant analysis. The discriminant analysis developed predictive discriminant functions for each value of recruitment, which was applied to all years during the same execution of the model. The model used a priori classification, and prior probabilities were computed from group sizes. Discriminant analysis also can be used to classify unknowns. To this end, I determined probability of recruitment in an average year by including long‐term mean rainfall at the Casa Grande weather station for 1981–2009 and for the period of record (1898–2009) (http://www.wrcc.dri.edu/cgi-bin/cliMAIN.pl?az1306).

## 3 RESULTS

### 3.1 Effect of moisture on survival and condition of single queens in the laboratory

**FIGURE 1:**
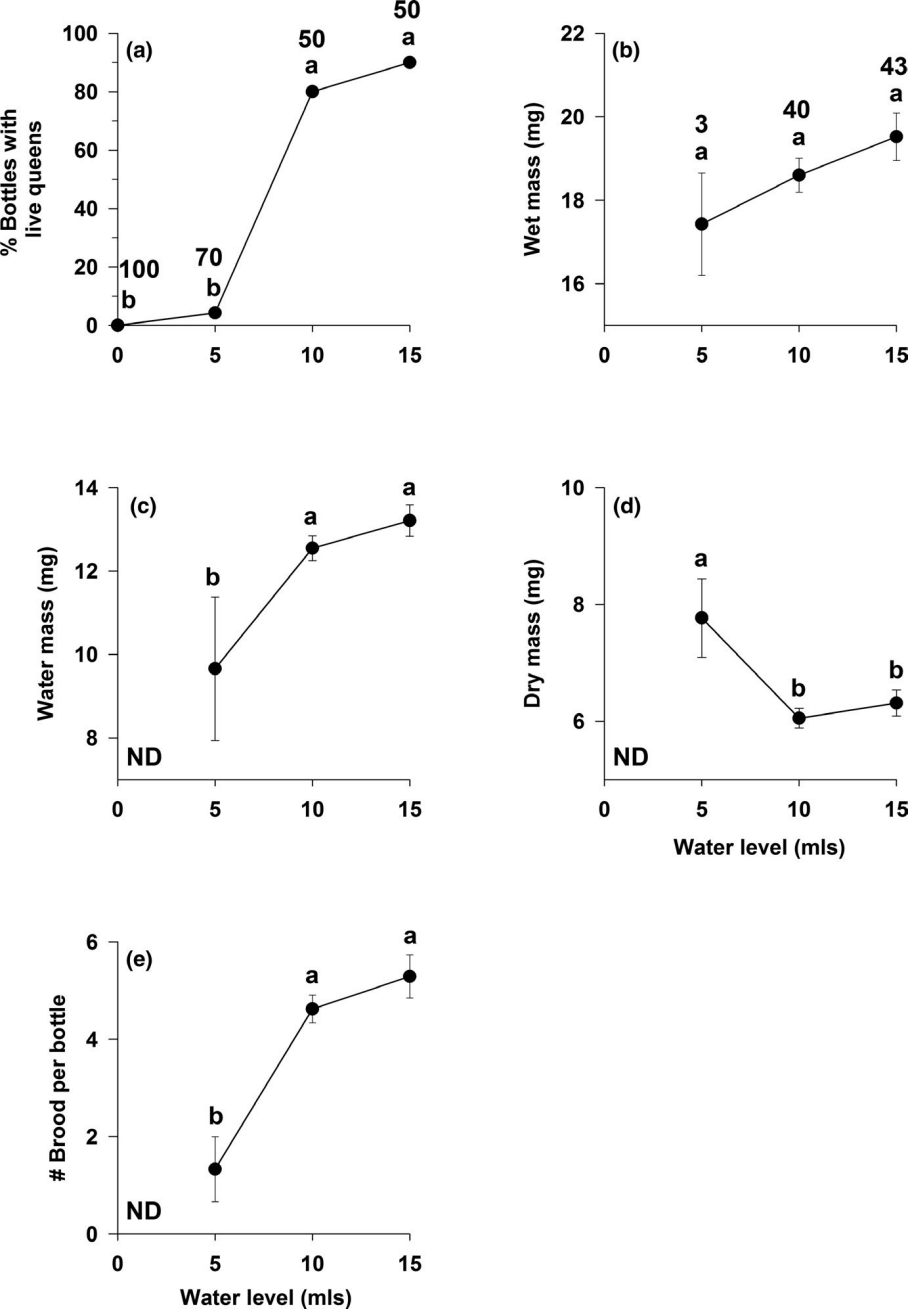
Effect of water level on: (A) queen survival, (B) queen wet mass, (C) queen water mass, (D) queen dry mass, and (E) number of brood (larvae, pupae, and workers) produced by single queens of the seed‐harvester ant *Veromessor pergandei*. Values are means + 1 *SE*. Within each panel, significant differences across moisture levels are indicated by the letters a‐b: a > b. Groupings are based on (A) a Wald chi‐square statistic (see text) or (B–E) a one‐way ANOVA followed by Duncan's multiple range test. Note that the one‐way ANOVA in panel D was not significant (*p* = 0.08), while Duncan's multiple range test was significant (*p* < 0.05). Initial sample size for each treatment is given in panel A; number of queens that survived to the end of the experiment (panels B–E) is given in panel B. ND = no data as no queens survived this treatment

Moisture level was positively associated with survival, measures of condition, and brood production for single queens. Queen survival was significantly higher in the highest moisture treatments (10 and 15 ml water) (logistic regression: *χ* ^2^ ≥ 31.5, *n* = 270 queens, *p* < 0.0001; Figure 1A), with survival reaching 90% in the 15 ml treatment. Queen wet mass (mg) did not vary across the three moisture levels (no queens survived in the 0 ml treatment) (one‐way ANOVA, *F*_2,83_ = 1.2, *p* > 0.29; Figure 1B). The two constituent parts of wet mass, water mass and dry mass, displayed converse patterns. Water mass varied by treatment (one‐way ANOVA, *F*_2,83_ = 4.0, *p* < 0.03), and was significantly lower for queens in the 5 ml than in the 10 ml and 15 ml treatments (Duncan's multiple range test, *p* < 0.05; Figure 1C). Dry mass did not vary by treatment (*F*_2,83_ = 2.6, *p* = 0.08), but Duncan's multiple range test was significant and showed that queens in the 5 ml treatment were heavier than those in the 10 ml and 15 ml treatments (Figure 1D). Total number of brood (workers, pupae, and larvae) also varied by moisture level (one‐way ANOVA, *F*_2,85_ = 4.6, *p* < 0.014), with the fewest brood produced in the 5 ml treatment (Duncan's multiple range test, *p* < 0.05; Figure 1E).

### 3.2 Effect of moisture and queen number in the laboratory

**FIGURE 2:**
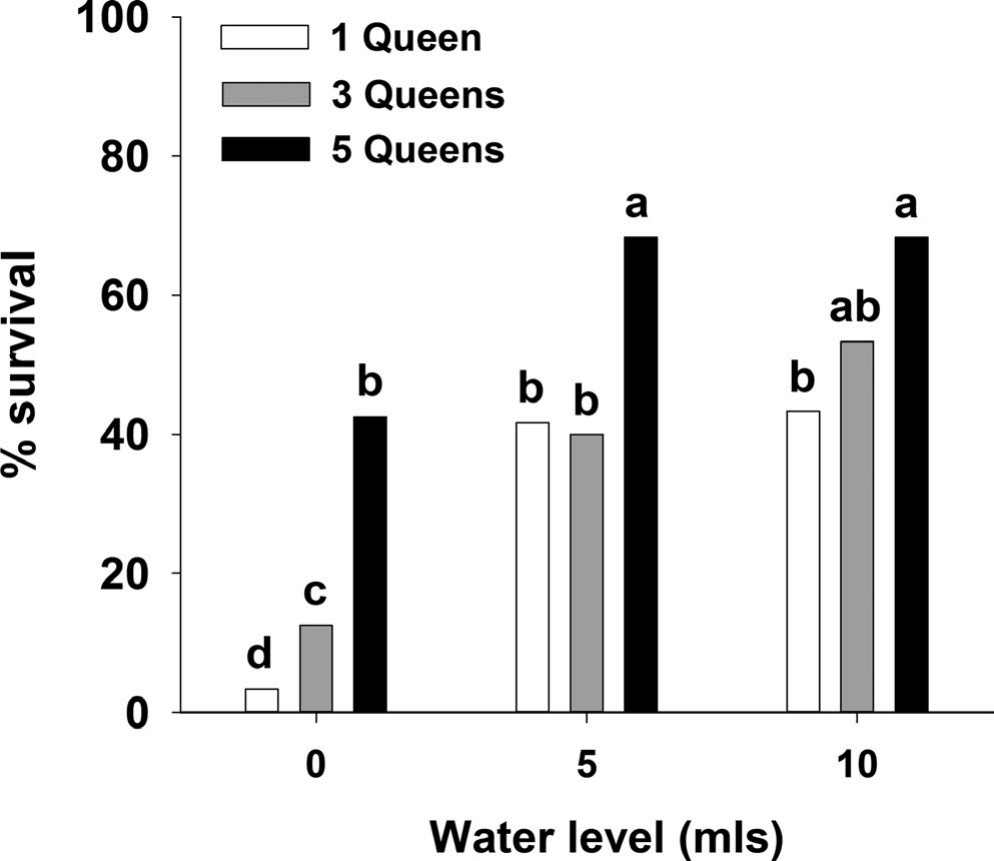
Percent survival for queens of the seed‐harvester ant *Veromessor pergandei* in the two‐factor bottle experiment (three queen numbers by three water levels). Significant differences across treatment cells are indicated by the letters a‐d: a > b > c > d. Groupings are based on the Wald chi‐square statistic (see text). Sample size per treatment cell; *n* = 120 queens for 0 and 5 mls water, *n* = 60 queens for 10 mls water

In the two‐way experiment, queen survival was positively influenced by both queen number and water level (Figure 2). Strength of the grouping effect can be assessed across water levels using the maximum Wald chi‐square value for comparisons across queen number within each water level. As predicted, grouping had the highest effect on queen survival in the 0 ml treatment, and its effect decreased progressively at higher water levels (*χ* ^2^_[maximum]_ 0 ml = 32.1, 5 ml = 18.8, 10 ml = 7.4). The 0 ml treatment was the only water level in which queen survival increased significantly from 1 to 3 to five queens (Figure 2).

**FIGURE 3:**
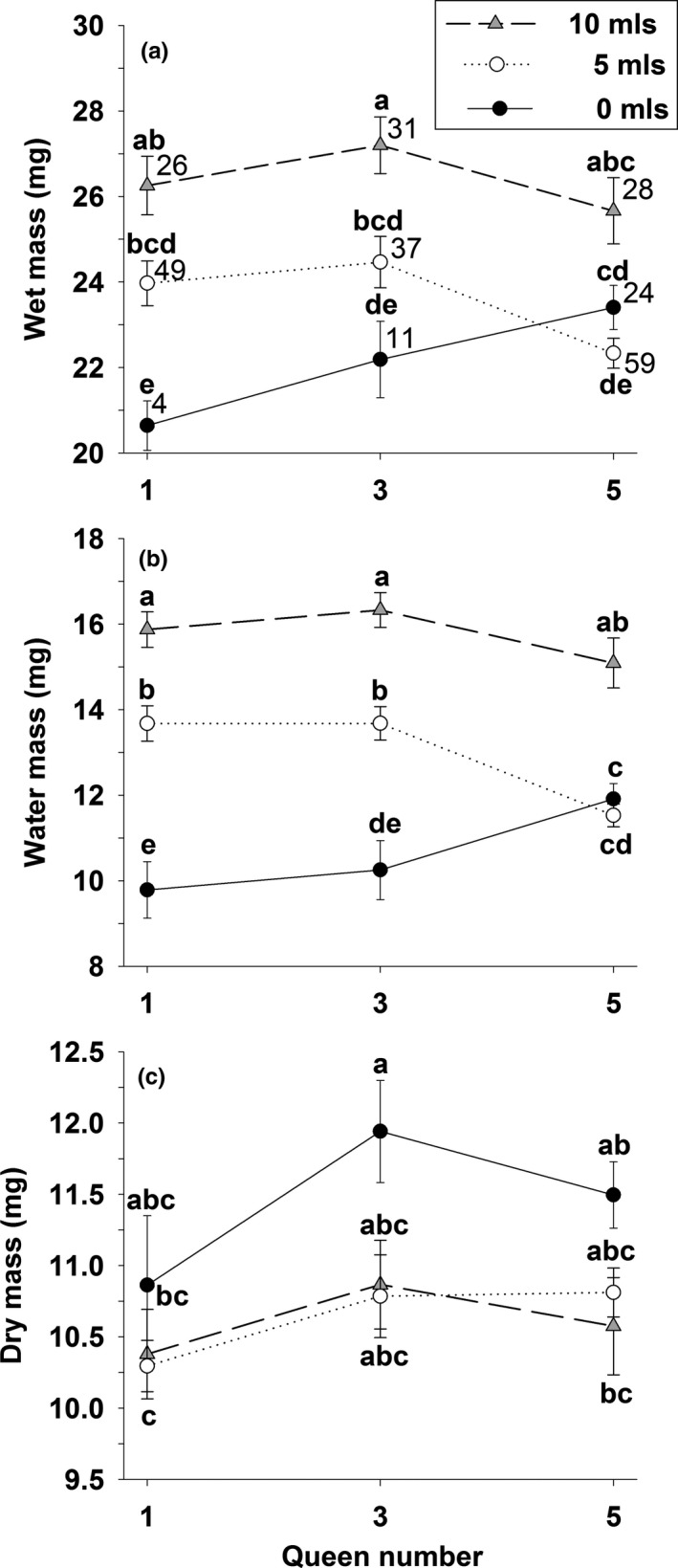
Effect of number and water level on: (A) wet mass, (B), water mass, and (C) dry mass for queens of the seed‐harvester ant *Veromessor pergandei* that survived in the two‐factor (queen number × water level) bottle experiment (see also Table 1). Values are means + 1 *SE*. Significant differences across treatment cells are indicated by the letters a‐e: a > b > c > d > e. Groupings are based on a one‐way ANOVA followed by Duncan's multiple range test. Panel A gives sample size for treatment cells in all panels. Data include only bottles in which > 2 or > 4 queens survived in the 3 and 5 queen bottles, respectively

In the two‐way ANOVA, water level was a significant effect for all three measures of queen condition (wet mass, water mass, dry mass), whereas queen number was not significant for any of these same measures (Table 1). The interaction term (water level × queen number) was significant for water mass, but not for wet mass or dry mass (Table 1). In post hoc tests for water level, wet mass at the end of the experiment was higher in the 10 ml treatment than in the 0 or 5 ml treatments (Duncan's multiple range test, *p* < 0.05), water mass increased progressively from the 0 to 5 to 10 ml treatments (*p* < 0.05), and dry mass was significantly higher in the 0 ml water level than in the 5 and 10 ml treatments (*p* < 0.05), presumably because queens in the latter two treatments invested more mass to produce brood (see Figures 1 and 2). The one‐way ANOVA displayed a similar pattern in that wet mass and water mass were consistently higher for the 10 ml treatment (Figure 3A,B), whereas dry mass was consistently higher in the 0 ml treatment (Figure 3C). All three variables displayed an interaction for three and five queens at the 10 and 15 ml treatments.

**TABLE 1:** Results of two‐way ANOVA for the effect of water level and queen number (independent variables) on wet mass (mg), water mass (mg), and dry mass (mg) (dependent variables) for queens of *Veromessor pergandei* in the bottle experiment (see text)

Source	*df*	Wet mass.		Water mass.		Dry mass.	
		*F*	*p*	*F*	*p*	*F*	*p*
Corrected model	8	8.5	**<0.001**	20.8	**<0.001**	2.5	**0.013**
Moisture level	2	24.2	**<0.001**	58.3	**<0.001**	3.2	**0.044**
Queen number	2	1.3	0.26	0.9	0.42	2.1	0.13
Queen number × moisture level	4	2.3	0.064	5.9	**<0.001**	0.3	0.9
Error	260						
Total	269						
*R* ^2^		0.21		0.39		0.07	

Note

Analyses included only bottles in which ≥ 2 and ≥ 4 queens survived in the 3 and 5 queen bottles, respectively. A separate two‐way ANOVA was run for each dependent variable (see also Figure 3). Degrees of freedom (*df*), *F*, and *p* values are included. Significant *p* values are in **bold** font.

### 3.3 Growth of single queen colonies in the laboratory

Single queens produced their first minim workers after an average of 34 days at 30°C, and they produced up to 25 minim workers without food. After 90 days, colony size increased to an average of 117.0 ± 14.0 total brood (52.8 ± 6.2 workers; 64.3 ± 8.3 larvae and pupae, *n* = 20 queens); the largest colony contained 245 brood (102 workers, 143 larvae and pupae).

### 3.4 Desiccation of founding queens in the field

**FIGURE 4:**
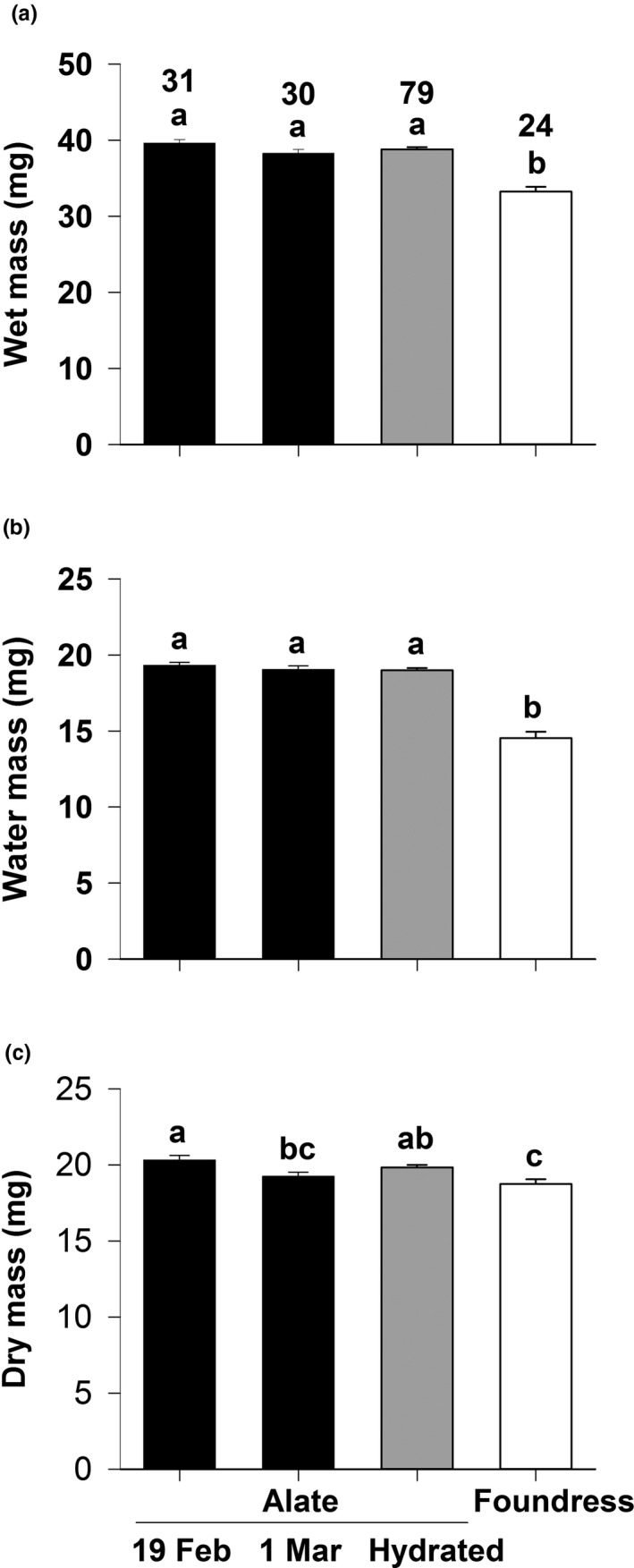
Effect of stage on: (A) queen wet mass, (B) queen water mass, and (C) queen dry mass for the seed‐harvester ant *Veromessor pergandei*. ■ = alate queens, ■ = hydrated alate queens, and □ = two‐week‐old founding queens. Values are means + 1 *SE*. Significant differences among the four groups are indicated by the letters a‐c: a > b > c. Groupings are based on a one‐way ANOVA followed by Duncan's multiple range test. Sample size is given in the top of panel A

Wet mass and water mass varied in a similar pattern across the four groupings of queens. For both measures, the three groups of alate queens collected from their natal nest (field‐weighed on 19 Feb, field‐weighed on 1 March, hydrated) were significantly heavier than foundress queens (one‐way ANOVA, *F*_3,160__[wet mass]_=30.0, *p* < 0.001; *F*_3,160__[water mass]_ = 67.6, *p* < 0.001; Duncan's multiple range test, *p* < 0.001; Figure 4A,B). Dry mass also varied across the four groupings (*F*_3,160__[dry mass]_ = 6.1, *p* = 0.001), and was lowest for foundress queens (*p* < 0.05; Figure 4C). Overall, wet mass of foundress queens averaged 5.62 mg (14.4%) less than that of alate queens. The two components of wet mass, water mass and dry mass, accounted for 4.55 mg (81.0%) and 1.07 mg (19.0%) of the lower founding queen mass, respectively. The 4.55 mg of water equated to 23.8% of the water mass of alate queens.

In the dehydration experiment, alate queens lost an average of 8.49 ± 0.17 mg of their body water at the point of mortality, which equated to 41.9 ± 0.5% of their hydrated water mass. This percentage was not correlated with initial queen wet mass (linear regression, *p* > 0.50). However, amount of water lost at the point of mortality was correlated with initial wet mass (linear regression: *Y* = 0.25*x* − 1.47, *F*_1,36_ = 31.4, *p* < 0.001, *R* ^2^ = 0.47), indicating that larger queens may better withstand desiccation.

These data can be extrapolated to the field‐collected foundress queens. Over the 12 days from their mating flight to excavation, field‐weighed foundress queens lost approximately 53.5% (4.55/8.49 mg) of the water that can be lost prior to mortality. Assuming a linear rate of water loss (see Johnson et al., 2011), founding queens of *V. pergandei* can survive desiccating field conditions for approximately 22.4 days.

### 3.5 Rainfall and recruitment in the field

Rainfall was a significant predictor for recruitment of new colonies over the 20 years of field data for both total rainfall (Wilks' *λ* = 0.54, Chi‐square _1_ *_df_* = 10.7, *p* = 0.001; Box's *M* test: *F*_1,228_ *_df_* = 0.37, *p* = 0.54) and for early and late rainfall (Wilks' *λ* = 0.52, Chi‐square test _2_ *_df_* = 11.1, *p* = 0.004; Box's *M* test: *F*_3,382_ *_df_* = 1.62, *p* = 0.18). The positive standardized canonical discriminant function coefficients for total rainfall (1.000) and for early and late rainfall (0.777 and 0.645) indicated that rainfall positively affected recruitment for both variables; that is, increased rainfall increases recruitment. The similar coefficients for early and late rainfall indicated that both periods had a similar effect on recruitment. Both early and late rainfall were significant in the stepwise discriminant analysis, and both variables had a tolerance of 1.0, indicating no overlap in the proportion of variance accounted for by the other variable. The robust nature of the discriminant model was evidenced by the high posterior probability for correctly predicting recruitment outcome (>0.90) in 14 of the 20 years for both total and for early and late rainfall (Table 2).

**TABLE 2:** Deviation from mean total rainfall (January–June) and early (January–March) plus late rainfall (April–June) (see text)

Year	Total rainfall (mm)	Early rainfall (mm)	Late rainfall (mm)	Recruitment/posterior probability
**1991**	**+31.8**	**+41.7**	**−9.9**	**NO (0.824; 0.901)**
**1992**	**+150.1**	**+97.8**	**+52.3**	**YES (0.992; 0.998)**
1993	+44.2	+60.2	−16.0	NO (0.706; 0.870)
1994	−3.1	−1.8	−1.3	NO (0.968; 0.970)
1995	+6.3	+18.0	−11.7	NO (0.948; 0.970)
1996	−50.9	−35.6	−15.3	NO (0.997; 0.998)
1997	−33.8	−33.3	−0.5	NO (0.994; 0.992)
1998	+70.5	+81.0	−10.5	YES (0.630)
				NO (0.602)
1999	−49.5	−58.9	+9.4	NO (0.997; 0.994)
2000	−19.9	−6.1	−13.8	NO (0.987; 0.992)
**2001**	**+60.2**	**+35.6**	**+24.6**	**NO (0.506)**
				**YES (0.657)**
**2005**	**+74.1**	**+72.4**	**+1.7**	**YES (0.673; 0.572)**
2012	−30.1	−24.0	−6.1	NO (0.992; 0.993)
2013	0.0	+11.2	−11.2	NO (0.962; 0.977)
2014	−60.0	−43.2	−16.8	NO (0.998; 0.999)
2015	+22.9	−8.1	+31.0	NO (0.883; 0.694)
2016	−2.6	−9.4	+6.8	NO (0.967; 0.956)
2017	−32.6	−17.6	−15.0	NO (0.993; 0.996)
2018	−53.2	−41.2	−12.0	NO (0.998; 0.998)
2019	+35.8	+47.6	−11.8	NO (0.790; 0.891)
1981–2009	0.0	0.0	0.0	NO (0.962; 0.963)
1898–2009	0.0	0.0	0.0	NO (0.974; 0.976)

Note

Deviation (mm) was calculated as the difference from the 1981–2009 mean rainfall from the weather station nearest the field site (Jan–Mar = 64.0 mm; Apr–June = 16.8 mm). Lines in **bold** font are years in which recruitment by *Veromessor pergandei* was observed; lines in normal font are years in which recruitment was not observed. The right column gives the discriminant casewise prediction (recruitment or no recruitment) and posterior probability for being in that group (total rainfall; early and late rainfall). Years with casewise predictions on two lines (1998, 2001) are those in which the model predicted differed recruitment outcomes for total rainfall and for early and late rainfall (total in first line; early and late on second line). The bottom two lines give predicted recruitment probabilities for the last 30 years (1981–2009) and for the period of record for the weather station nearest the field site (1898–2009).

For total rainfall, the discriminant model correctly predicted recruitment outcome in 17 of 20 years (85.0%), with the two years in which the model correctly predicted recruitment (1992, 2005) receiving the highest total rainfall (>74.0 mm above the long‐term mean for both years) (Table 2). For early plus late rainfall, the discriminant model correctly predicted recruitment outcome in 19 of 20 years (Table 2), with the three years in which the model correctly predicted recruitment (1992, 2001, 2005) being the only three years in which both early and late rainfall were higher than the long‐term mean (Table 2; Appendix S1). The two years (1991, 1998) in which one or both models incorrectly predicted recruitment showed the importance of timing of rainfall. Both years received higher than average total rainfall, with an abundance of early rainfall that included significant rainfall at the end of March, which saturated soils for early parts of the late season (Table 2). Recruitment occurred in 1991 because a significant mid‐June rainfall allowed queens to rehydrate and reset their physiological clock, whereas late rainfall in 1998 was limited to early April followed by 11 weeks of drought, and recruitment did not occur.

Importance of high rainfall affecting recruitment also was demonstrated by the discriminant model predicting no recruitment for the two ungrouped periods (1981–2009 and 1898–2009), with a posterior probability > 0.95 for both total and for early plus late rainfall (Table 2, Appendix S1). Differences between the total rainfall and the early and late rainfall models were caused, in part, by a lack of correlation between early and late rainfall over the years of study (*p* > 0.20, *n* = 20).

## 4 DISCUSSION

### 4.1 Effects of moisture on queen survival and condition

That water is the most important factor affecting survival and establishment of *V. pergandei* colonies is demonstrated by field data and laboratory experiments. Field data on foundress water balance were collected during 1999, in which early rainfall was far below average (Table 2). During this period, alate queens in their natal nests remained fully hydrated (Figure 4), whereas founding queens desiccated significantly within 2 weeks of initiating their nest because the top 0.5 m from the soil surface, that is, those areas used by founding queens, remain desiccating except for short intervals following rainfall (Pantastico‐Caldas & Venable, 1993; Reynolds et al., 2004; Young & Nobel, 1986). Desiccation is undoubtedly the most significant source of queen mortality in most years given that extended dry periods often follow mating flights, and that areas near the soil surface become warmer and drier from early April to late June. That desiccation does not affect mature colonies of *V. pergandei* is demonstrated in that their alate queens remained hydrated even in an exceptionally dry year. The mechanism by which colonies obtain water is unknown, but incipient colonies likely experience moisture limitation through at least the first year, until the nest is deep enough and/or sufficient workers are present to buffer environmental harshness and access water as do mature colonies. Colonies of *V. pergandei* also produce significantly fewer sexual reproductives in dry than in wet years, providing an additional mechanism to limit recruitment in drier years (Cahan, 2001b; Ryti & Case , 1988).

Alternatively, survival, condition, and brood production by single queens of *V. pergandei*, and survival and condition of pleometrotic queens consistently increased at higher water levels. That moisture commonly affects survival and establishment of desert ants is suggested by similar effects of water level on survival, queen condition, and brood production in two other common desert seed‐harvester ants (*Pogonomyrmex barbatus*, *P. rugosus*) (Johnson, 1998). An additional parallel between the two studies was that queen dry mass decreased at higher moisture levels, reflecting the energetic cost of rearing more workers and brood.

### 4.2 Pleometrosis and colony survival in *V. pergandei*

Both higher water levels and increased queen number enhanced queen survival in the two‐factor bottle experiment. That queen survival was most enhanced in grouped compared to solitary queens in the driest treatment supports the desiccation resistance hypothesis (Figure 2). However, this result contrasted with the main effects of the two‐way ANOVA, which indicated water level was a significant effect for all three queen condition variables (wet mass, water mass, dry mass), whereas queen number was not significant for any of these variables (Table 1). These results parallel those of a similar experiment with groups of one and three *V. pergandei* queens in which grouping increased survival in the driest treatment, but had no effect at higher water levels. Queen condition (proportional water content) and number of brood produced (larvae, pupae, workers) also increased with moisture level, but queen number was not significant (Cahan, 1999).

The interaction term between moisture level and queen number was significant in both studies, indicating that water mass of solitary queens was lower than that of grouped queens at low moisture levels and that these differences disappeared at higher moisture levels (Figure 3).

The mechanism by which queen grouping enhances water balance under desiccating conditions is unknown. Grouping decreases water loss in insects by decreasing surface area and/or increasing local humidity, and the advantages generally increase with group size (Klok & Chown, 1999; Rasa, 1997). Queens also place their brood in one aggregate pile, which could decrease water loss by decreasing surface area such as occurs for egg masses of butterflies (Clark & Faeth, 1998), and the benefit would likely increase with number of brood, that is, number of queens. Lastly, strongly desiccating conditions probably result in the queens cannibalizing other queens or brood to regain moisture and/or nutrition (see Cahan, 1999; Tschinkel, 1993).

These results support the desiccation resistance hypothesis for *V. pergandei* in the narrow sense because grouping benefited both survival and water balance of queens, but only under desiccating conditions (see also Cahan, 1999). However, the desiccation resistance hypothesis does not explain the evolution of pleometrosis in the broad sense because these benefits occurred only under the driest conditions, that is, those in which queens have the most negative water balance, produce the fewest brood, and in which recruitment does not occur in the field. It is also doubtful that desiccated queens would experience sufficient rainfall after early April to rehydrate and produce sufficient brood to survive under the increasingly adverse conditions that occur in the ensuing 2–3 months prior to summer rains. That grouped queens also displayed enhanced survival compared to single queens in other studies suggests involvement of mechanisms such as social facilitation rather than desiccation resistance (see also Johnson, 2004; Waloff, 1957).

Overall, these data suggest that pleometrosis in *V. pergandei* evolved as a mechanism to enhance establishment of new colonies in areas with harsh abiotic (desiccating) conditions, facilitating colonization of habitats in which solitary queens could not persist (see also Avilés, 1999; Sun et al., 2014). Under this scenario, pleometrosis is advantageous only in favorable (wet) years when recruitment occurs, but its effect is negligible in unfavorable (dry) years when recruitment would not occur for single or pleometrotic queens (see also Raczkowski, 2003). This favorable‐year hypothesis supports enhanced worker production as the primary advantage of pleometrosis because faster growth facilitates colonies more quickly escaping harsh conditions near the soil surface (see also Raczkowski, 2003). In this sense, queen number is an indirect rather than direct benefit of pleometrosis (Table 1); that is, in favorable (wet) years, one queen from a pleometrotic association survives queen reduction, whereas haplometrotic queens produce too few workers to survive even in wet years. That the evolution of pleometrosis is linked to more desiccating conditions also is supported by the transition zone to pleometrosis correlating with reduced precipitation, decreased vegetative biomass, and lower colony density (Cahan, 2001a, 2001b; Cahan et al., 1998).

Ecological constraints such as intense intraspecific competition for space or other resources usually are presumed to select for grouping/cooperation in taxa that include ants, some other social insects, and birds (Bernasconi & Strassman, 1999; Brockman, 1997). More recently, it was hypothesized that cooperative breeding in many bird species represents a mechanism to colonize harsh environments (Cockburn & Russell, 2011; Cornwallis et al., 2017; Jetz & Rubenstein, 2011; Shen et al., 2017); similar suggestions have been made for pleometrosis in *V. pergandei* (Cahan, 1999; Pfennig, 1995). This dichotomy suggests that cooperation evolves in benign, stable environments where intraspecific competition is intense, as well as in harsh environments where temperatures are hot and interannual variation in rainfall is high (Shen et al., 2017). In this study, *V. pergandei* queens experience harsh abiotic environments with high interannual variation in rainfall, supporting the hypothesis that pleometrosis evolved to colonize harsh habitats. One measure of environmental harshness is the coefficient of variation in rainfall across years (standard deviation/mean) (see also MacMahon & Wagner, 1985), which is generally inversely correlated with absolute amount of rainfall. At this site, the average January–June rainfall from 1981–2009 was 80.77 mm with a coefficient of variation at 0.64.

That harsh environments favor pleometrosis in *V. pergandei* can also be examined using predictions of per capita reproductive output derived from insider–outsider conflict theory (Shen et al., 2017). This theory predicts two categories of grouping benefits that relate to benign, stable and harsh environments, respectively—(a) resource defense benefits that derive from groups defending critical resources, and (b) collective action benefits that result from social cooperation among group members. Per capita reproductive output is predicted to decrease with increasing group size for resource defense benefits, whereas per capita reproductive output increases for collective action benefits. Several pleometrotic ants such as *Solenopsis invicta* and *Lasius niger* display decreases in per capita production of minim workers as queen number increases (Bernasconi & Keller, 1998; Sommer & Hölldobler, 1995; Tschinkel, 1993), presumably because of intense intraspecific competition. Alternatively, *V. pergandei* inhabits harsh desert habitats and number of minims produced per queen remains the same, at least up to five queens (Rissing & Pollock, 1991). Thus, observed variation in per capita minim production across ant species also might explain their inhabiting benign, stable versus harsh environments.

In *V. pergandei*, rapid colony growth is achieved by one queen producing up to 25 first brood workers, which is significantly more than produced by sympatric species of *Pogonomyrmex* (Johnson, 1998). Subsequent growth leads to some colonies containing > 200 workers and brood within 3 months (this study; C. Kwapich, unpub. data). Number of workers produced in the first brood increases linearly with queen number (Rissing & Pollock, 1991; this study), suggesting that pleometrotic colonies can contain > 1,000 workers within several months. Indeed, field colonies initiated in March sometimes contain several hundred workers by the time of the census for new colonies (late August to late November) (R.A. Johnson, pers. obs.), such that these colonies had survived the driest and hottest months of the year and were entering the more moderate fall and winter seasons, as well as beginning the ergonomic (growth) phase in which survival rate increases dramatically (Cole, 2009).

The several other proximate hypotheses suggested to explain the evolution of pleometrosis in *V. pergandei* (see introduction) likely can be discounted. The brood raiding and foraging success hypotheses both predict that the advantage of pleometrosis results from colonies containing more workers. Brood raiding is viewed as a means to gain a competitive advantage in inter‐colony competition (Bernasconi & Strassman, 1999; Rissing & Pollock, 1987, 1991; Tschinkel, 1992), and it likely functions similarly for *V. pergandei*, except that the advantages would occur only during favorable (wet) years when recruitment occurs. This hypothesis was criticized by Brown (2000) because brood raiding between incipient colonies had not been observed in the field (see also Pfennig, 1995), but recent observations document brood raiding in the field (Raczkowski, 2003; R.A. Johnson, pers. obs.). Moreover, I propose that brood raiding increases colony size and thus functions in a manner similar to pleometrosis, except that workers are added from neighboring colonies rather than from workers produced by additional queens. The foraging success hypothesis (Brown, 2000) predicts that the additional workers in pleometrotic colonies increase foraging success and hence colony survival and growth. I discount this hypothesis because of the high energetic gain from harvesting seeds, which is more than 100 times the energy cost per trip (Weier & Feener, 1995), and because foraging success would already be high given that recruitment occurs only in favorable (wet) years when production and availability of annual seeds are highest (Beatley, 1974; Rosenzweig, 1968).

Pfennig (1995) also suggested that pleometrosis might result from newly mated queens needing to quickly obtain cover underground in their incipient nest so as to decrease desiccation and exposure to predators. Mated queens undoubtedly lose water before entering a nest, but this is an ephemeral effect (1–2 hr) that would exert weak selection pressure given the low water loss rate and high desiccation tolerance exhibited by *V. pergandei* queens (Johnson, 2000d; Johnson et al., 2011; this study), combined with the fact that mating flights occur during cooler months, that is, February–March. Likewise, quickly entering a nest does not preclude predation because centipedes and other predators commonly invade incipient nests and kill all queens (C. Kwapich, pers. comm.).

Two field studies have examined pleometrosis in *V. pergandei* with contradictory results. Pfennig (1995) suggested that interference competition among incipient colonies does not select for pleometrosis because: (a) there was no difference in survival or longevity for one versus two queen colonies, (b) foundresses initiated colonies near adult colonies, and (c) number of queens in incipient colonies increased with distance from adult colonies. Alternatively, at the same site Raczkowski (2003) found higher survival and greater longevity for three queen compared to one queen colonies, and that distance to a mature colony did not affect survival or longevity.

Both field studies demonstrate that very low survival in the short and especially longer term requires experiments that use large sample sizes (see also Cole & Wiernasz, 2002) in multiple years (including wet years) and/or manipulate soil moisture. Note however that experiments that manipulate soil moisture often fail because locally wet soils attract fire ants (*Solenopsis xyloni*) that kill the foundresses. Another caveat is that pleometrotic colonies of *V. pergandei* regularly contain 3–7 or more queens (Cahan et al., 1998; Pollock & Rissing, 1985), such that both field experiments used few queens compared to the number found in typical associations.

Pleometrosis undoubtedly is a derived trait in *V. pergandei*, especially given that queens are haplometrotic throughout a large portion of the species range (Cahan, 1999; Helms & Helms Cahan, 2012; Johnson, 2000b). The first step in the evolution of pleometrosis necessitates that non‐aggressive alate queens (unmated alate queens in their natal nest) maintain their non‐aggressive behavior after mating, rather than becoming aggressive as occurs for species in which queens are haplometrotic. Thus, changes in gene expression and/or production of neurochemicals that increase aggression cannot occur (Helmkampf et al., 2016; Koyama et al., 2015; Muscedere et al., 2016; Ohkawara & Aonuma, 2016). If this same non‐aggressive behavior continues after minim workers emerge, then queens would remain cooperative, leading to primary polygyny. Alternatively, queen reduction after minim workers emerge requires a change in gene expression and/or production of neurochemicals that increase aggression.

### 4.3 Episodic recruitment

Water plays a critical role for foundress survival and brood production in *V. pergandei* given that colony recruitment occurs in only favorable, wet years. This pattern parallels that for many desert plants that exhibit episodic recruitment during years of high summer rainfall (Johnson, 2006; Johnson et al., 1993; Jordan & Nobel, 1979, 1981; Steenbergh & Lowe, 1969; Winkler et al., 2018), but this is the first case in which recruitment of a desert ant has been linked directly to rainfall.

Quantity and timing of rainfall affect recruitment of new colonies because recruitment only occurs in years in which both early and late rainfall well exceed the long‐term mean. Lack of regular recruitment is also supported by the discriminant analysis showing a near‐zero probability of recruitment in an average rainfall year based on the two long‐term ungrouped periods that used mean rainfall as a predictor. Early rainfall maintains hydration of foundresses and allows them to produce the maximum number of brood. However, these incipient colonies must survive increasingly hot and arid conditions over the next several months, and above average late rainfall rehydrates the queen and her workers, allowing the colony to reset their physiological “clock” for desiccation tolerance so that they can survive until summer rains (Johnson, 2006). For cactus, time to desiccation increases with plant volume (Jordan & Nobel, 1981; Nobel, 1988), and a similar study suggests that increased colony size buffers against desiccation induced mortality for ant queens (Kaspari & Vargo, 1995). A primary difference between plants and desert ants is that the effect of moisture should be more pronounced for ants than plants because seeds can remain dormant for several years until appropriate germination conditions occur, whereas ant queens “germinate” immediately, and hence cannot avoid inclement conditions in any one year (Johnson, 2006).

Overall, more studies need to examine the role of moisture in colony recruitment and desiccation tolerance in *V. pergandei* and other desert ants (see Menke et al., 2007). For example, the water source for mature colonies is unknown, but colonies remain hydrated given that workers lose water during foraging trips (Lighton et al., 1994), and that alate queens in their natal colony are hydrated under periods of extended drought while foundresses dehydrate under the same conditions (this study). Metabolic water is an unlikely source because it provides relatively little water for workers or queens of sympatric harvester ants (e.g., *Pogonomyrmex*). Additionally, these ants lack special adaptations to obtain water given that queens lose water even at very high humidities (97.5%) (Johnson, 1998, 2000a; Lighton & Feener, 1989). Colony size and depth are likely important in escaping desiccating conditions, but the source of water remains unclear even for mature colonies that extend several meters below the surface (see Tevis, 1958).

*Veromessor pergandei* also likely will be affected by climate change given that models predict increased aridity (both warmer temperatures and decreased winter–spring rainfall) for deserts of the southwestern United States that are already experiencing an extended drought (Walsh et al., 2014; Williams et al., 2020). Such changes are predicted to affect a greater degree of episodic recruitment in *V. pergandei*, resulting in fewer age classes and more irregular population dynamics. Changes in aridity might also change microdistribution pattern in *V. pergandei* and other ants. *Veromessor pergandei* inhabits locales with more coarse‐textured, sandy soils, and it becomes uncommon to absent in finer‐textured, clayey soils (Johnson, 1992). This microhabitat limitation appears to relate to water availability because clayey soils hold water more tightly than sandy soils such that the sandy soil has a greater water potential even at a lower water content (Nilsen & Orcutt, 1996; Reynolds et al., 2004). Thus, infrequent recruitment by *V. pergandei* in marginal, less sandy soils would be further reduced. Distribution restrictions based on rainfall also parallel desert plants given that some cacti inhabit only sites where rainfall affects seedling establishment in > 10% of the years (Jordan & Nobel, 1982). Changes in rainfall patterns might also affect ecological attributes of a species such as queen size. For example, Wiernasz and Cole (2003) found that larger queens of *Pogonomyrmex occidentalis* were significantly more likely to survive early stages of colony founding than were smaller queens, and such a pattern would likely be enhanced under increasing aridity. This leads to the hypothesis that one explanation for the evolution of pleometrosis is that it functions as a behavioral mechanism to increase total queen mass beyond that possible at the level of individual queens. More total reproductive energy facilitates producing more brood, which is imperative for establishing new colonies.

## CONFLICT OF INTEREST

The author declares that he has no competing interests.

## AUTHOR CONTRIBUTION

**Robert A. Johnson:** Conceptualization (lead); Data curation (lead); Formal analysis (lead); Funding acquisition (lead); Investigation (lead); Methodology (lead); Project administration (lead); Resources (lead); Software (lead); Supervision (lead); Validation (lead); Visualization (lead); Writing – original draft (lead); Writing – review and editing (lead).

## Supporting information

Appendix S1
