# Supplementary material for: Desiccation limits recruitment in the pleometrotic desert seed‐harvester ant Veromessor pergandei
Source: Ecol Evol. 2020 Nov 22;11(1):294–308. doi: 10.1002/ece3.7039 (PMC7790620; doi:10.1002/ece3.7039)
Supplement: Supplementary file 1 — Appendix S1 [file ECE3-11-294-s001.pdf]

Appendix 1. Weekly rainfall (mm) for January–June from the weather station nearest to the field site (see text). Panels with filled black bars are years in which recruitment was observed by *Veromessor pergandei*; panels with filled gray bars are years in which recruitment was not observed. The vertical dotted line separates January–March (early rainfall) from April–June (late rainfall; see text). Year is given in the top left of each panel; numbers on each side of the vertical line are deviation (mm) from the 1981–2010 average rainfall (Jan–Mar = 64.0 mm, Apr-June = 16.8 mm); the upper right gives the discriminant casewise prediction (recruitment or no recruitment) and posterior probability for being in that group (total rainfall; early and late rainfall). Long-term mean weekly rainfall (1981–2009) is shown in the last panel (white hatched bars); *P* values in the first line are for the period 1981–2009, in the second line for the period of record (1898-2009). Years in which casewise predictions are on two lines (1998, 2001) are those in which the model predicted differed recruitment outcomes for total rainfall and for early and late rainfall (total in first line, early and late rainfall on second line).

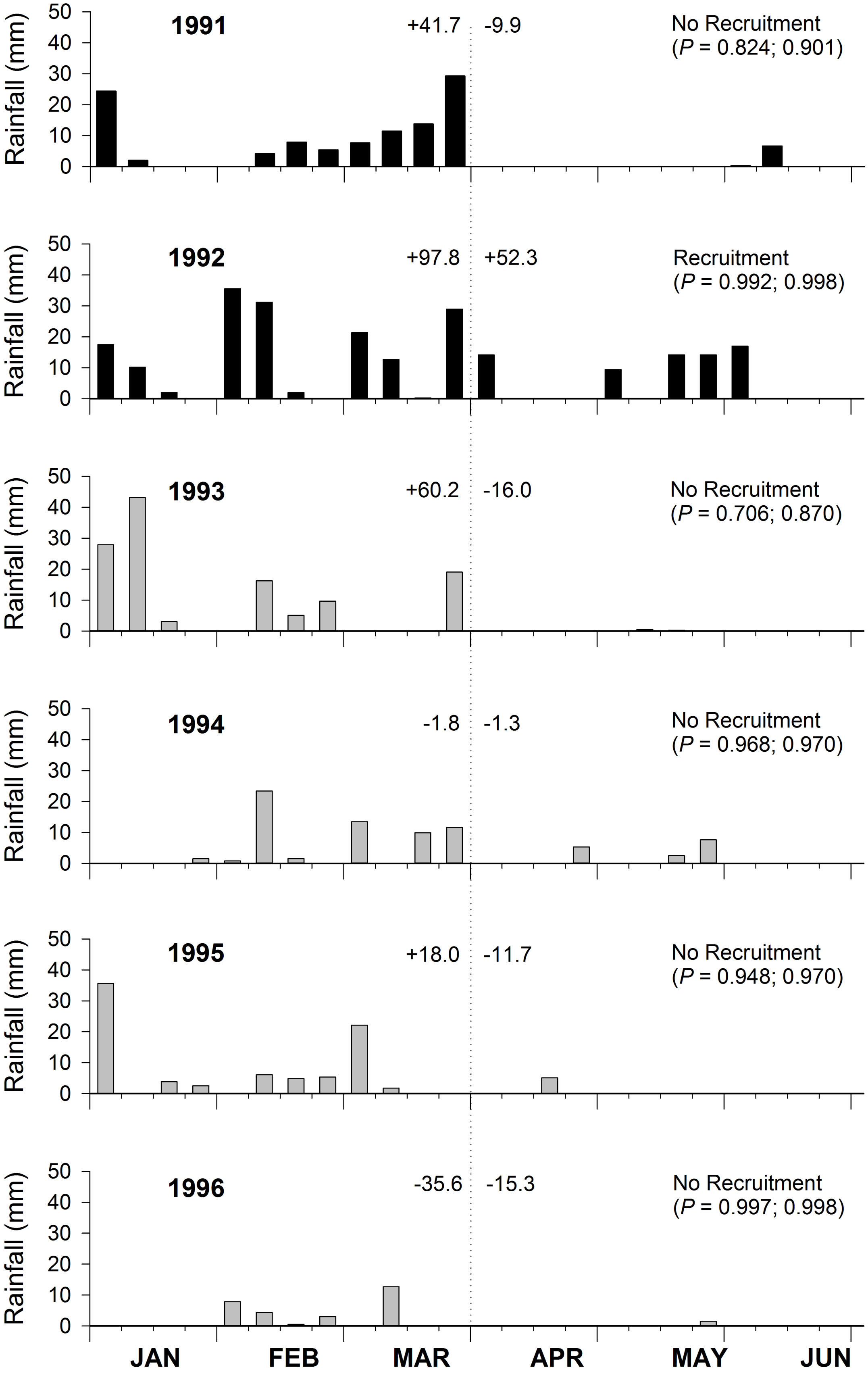

Rainfall (mm)
50
40
30
20
10
0
1991
+41.7
-9.9
No Recruitment
(P = 0.824; 0.901)
1992
+97.8
+52.3
Recruitment
(P = 0.992; 0.998)
1993
+60.2
-16.0
No Recruitment
(P = 0.706; 0.870)
1994
-1.8
-1.3
No Recruitment
(P = 0.968; 0.970)
1995
+18.0
-11.7
No Recruitment
(P = 0.948; 0.970)
1996
-35.6
-15.3
No Recruitment
(P = 0.997; 0.998)
JAN
FEB
MAR
APR
MAY
JUN

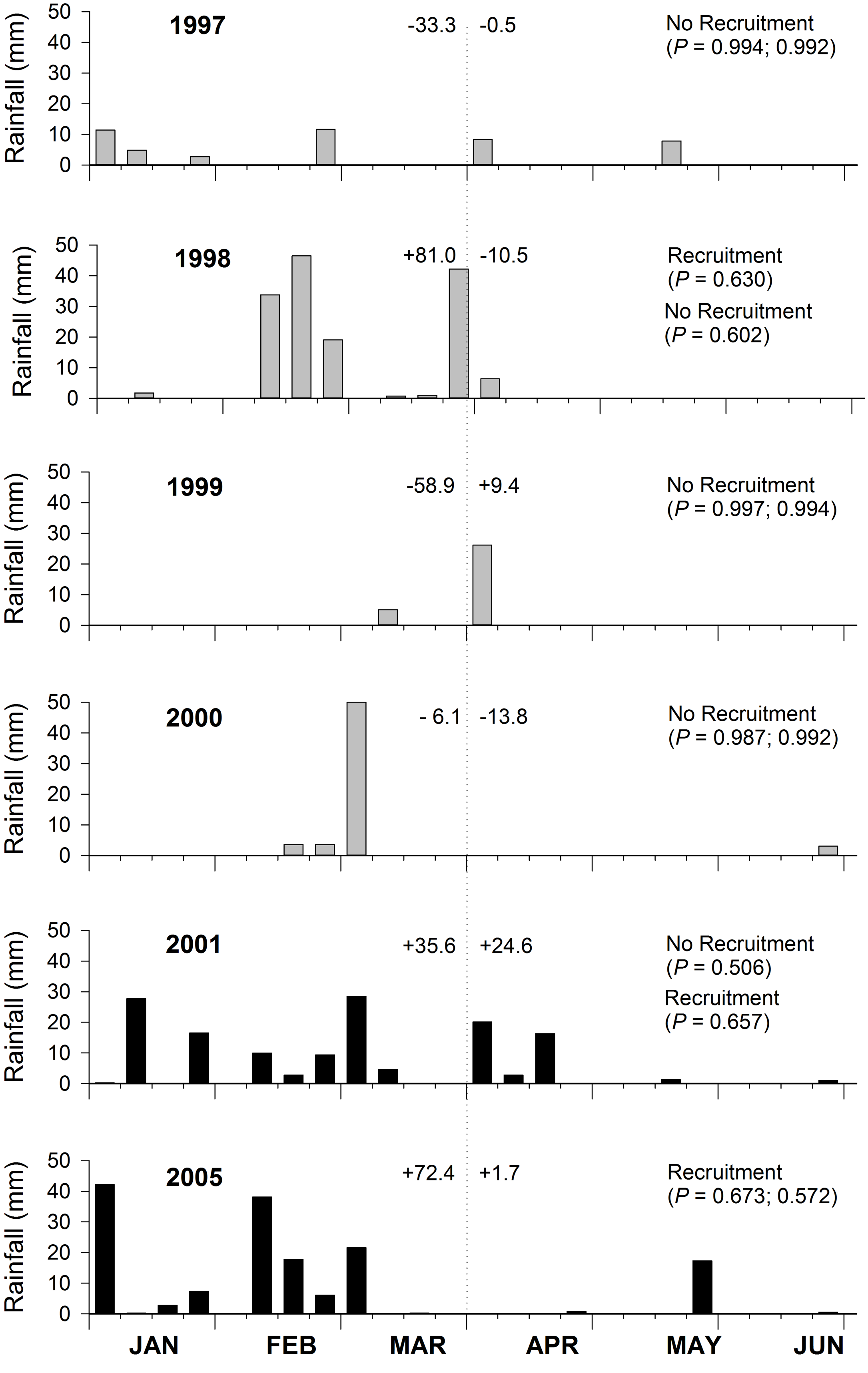

Rainfall (mm)
1997
-33.3
-0.5
No Recruitment
(P = 0.994; 0.992)
1998
+81.0
-10.5
Recruitment
(P = 0.630)
No Recruitment
(P = 0.602)
1999
-58.9
+9.4
No Recruitment
(P = 0.997; 0.994)
2000
- 6.1
-13.8
No Recruitment
(P = 0.987; 0.992)
2001
+35.6
+24.6
No Recruitment
(P = 0.506)
Recruitment
(P = 0.657)
2005
+72.4
+1.7
Recruitment
(P = 0.673; 0.572)
0
10
20
30
40
50
JAN
FEB
MAR
APR
MAY
JUN

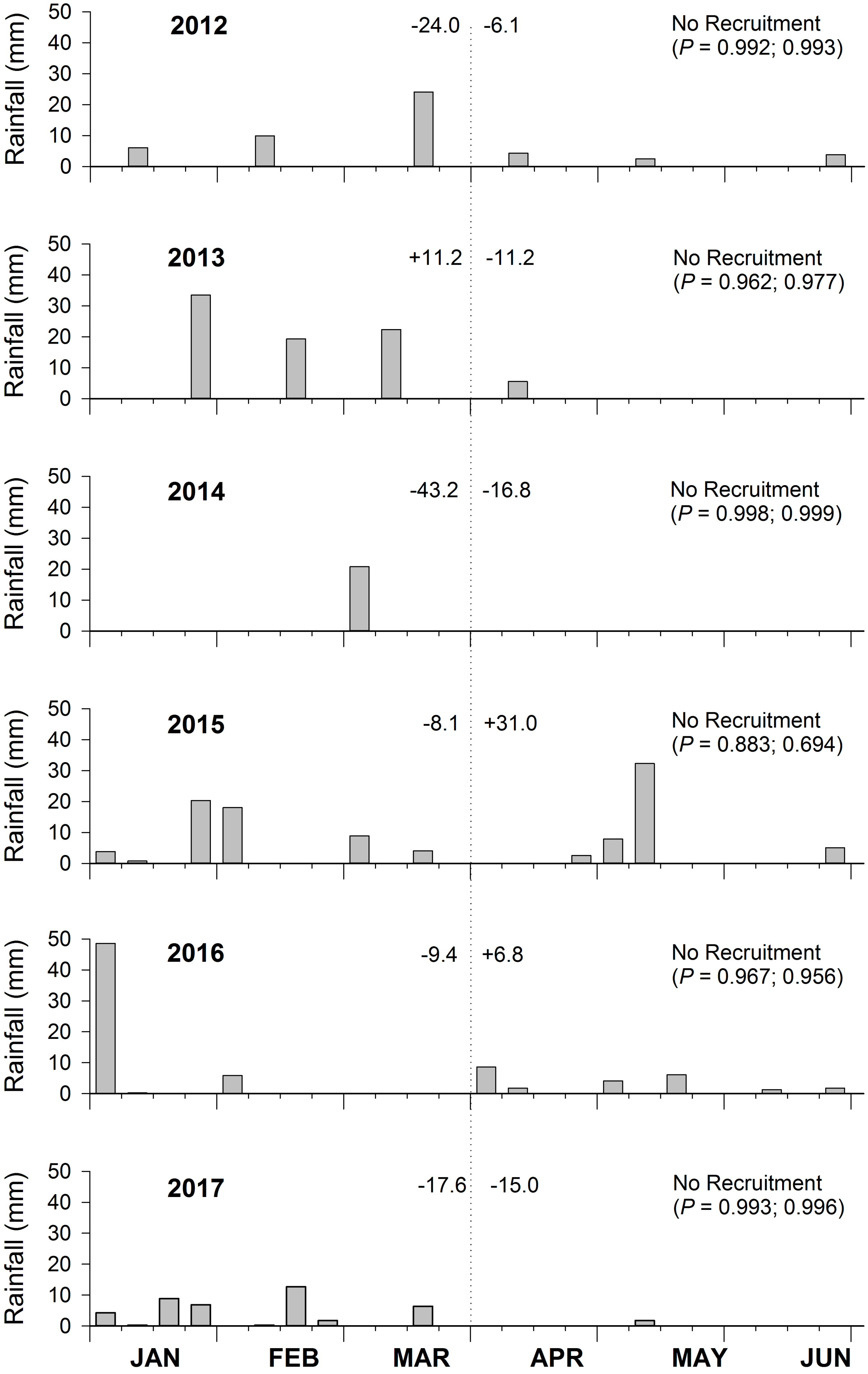

Rainfall (mm)
2012
-24.0
-6.1
No Recruitment
(P = 0.992; 0.993)
2013
+11.2
-11.2
No Recruitment
(P = 0.962; 0.977)
2014
-43.2
-16.8
No Recruitment
(P = 0.998; 0.999)
2015
-8.1
+31.0
No Recruitment
(P = 0.883; 0.694)
2016
-9.4
+6.8
No Recruitment
(P = 0.967; 0.956)
2017
-17.6
-15.0
No Recruitment
(P = 0.993; 0.996)
0
10
20
30
40
50
JAN
FEB
MAR
APR
MAY
JUN

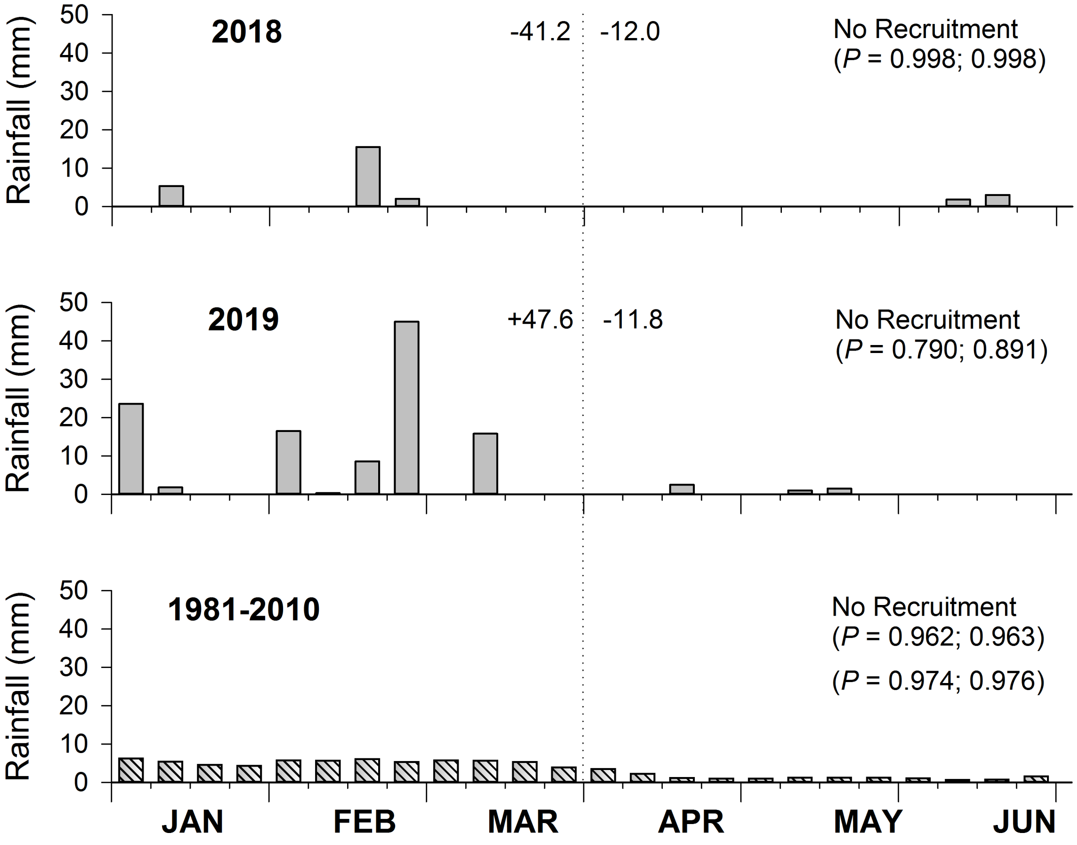

2018
-41.2
-12.0
No Recruitment
(P = 0.998; 0.998)
2019
+47.6
-11.8
No Recruitment
(P = 0.790; 0.891)
1981-2010
No Recruitment
(P = 0.962; 0.963)
(P = 0.974; 0.976)
Rainfall (mm)
50
40
30
20
10
0
JAN
FEB
MAR
APR
MAY
JUN
